# Neuronal microtubules impact lifespan

**DOI:** 10.18632/aging.102224

**Published:** 2019-09-06

**Authors:** Ellen Apple, Lizhen Chen

**Affiliations:** 1 Barshop Institute for Longevity and Aging Studies, Department of Cell Systems and Anatomy, UTHSCSA, San Antonio, TX 7822, USA

**Keywords:** microtubule stability, neuron integrity, longevity, lipid storage, daf-16

Microtubules (MTs) play fundamental roles in cellular functions including cell division, cell shape, and intracellular transport. Abnormal MT regulation has been linked to age-related disorders and diseases, and MTs often serve as targets for disease therapies. MT regulation is particularly important for neurons, since their polarized morphologies dictate heavy reliance on MT function for their maintenance. MT regulation is involved on several levels in neuronal function and maintenance of neuronal structure, and also appears to be a general downstream indicator and effector in age-dependent neurodegeneration [1]. The nervous system modulates lifespan in various species. It detects sensory cues from the environment and internal signals from the animal, and coordinates organismal metabolic homeostasis and energy balance [2,3]. Different neuron types respond to distinct cues to extend or shorten lifespan through activating distinct neuronal signals and signaling pathways, including the insulin/insulin-like growth factor-1 signaling (IIS) pathway [4,5], which plays an evolutionarily conserved role in regulating lifespan. Despite the essential role of MTs in neuronal function and the central role of the nervous system in regulating longevity, MT regulation has not been directly linked to lifespan modulation.

We have recently reported that mutations in MT regulators can affect lifespan in a DAF-16 dependent manner [6]. We found that loss of EFA-6, a protein promotes MT catastrophe [7], increased mean lifespan of *C. elegans* and delayed age-associated changes in neuronal integrity, such as axon blebbing and branching as well as mislocalization of synaptic vesicles to dendritic structures within touch sensory neurons [4]. The effects of the loss of EFA-6 opposed those effects seen with the loss of PTL-1, the worm homolog of human Tau protein that stabilizes MT, in previous research [8] as well as experiments conducted in our study [6]. These results suggest that shifting the balance of neuronal microtubule regulation towards stabilization rather than destabilization without completely abolishing microtubule destabilization processes facilitates the maintenance of neuronal structure and promotes organismal longevity in *C. elegans.* The *efa-6* loss-of-function mutants also maintained greater touch sensitivity and motor function during aging compared to wild-type worms. These differences in neuronal integrity and functional ability were time/aging-dependent with no significant differences between the efa-6 loss-of-function mutants and wild-type worms at day 1 of adulthood but significant differences between the groups by days 9 and 10 of adulthood [6]. In addition, the expression of EFA-6 in neurons, but not in muscle, rescued the phenotypic changes seen in the loss-of-function mutants, suggesting that the regulation of microtubules within neurons is what contributes to the regulation of lifespan.

**Figure 1:**
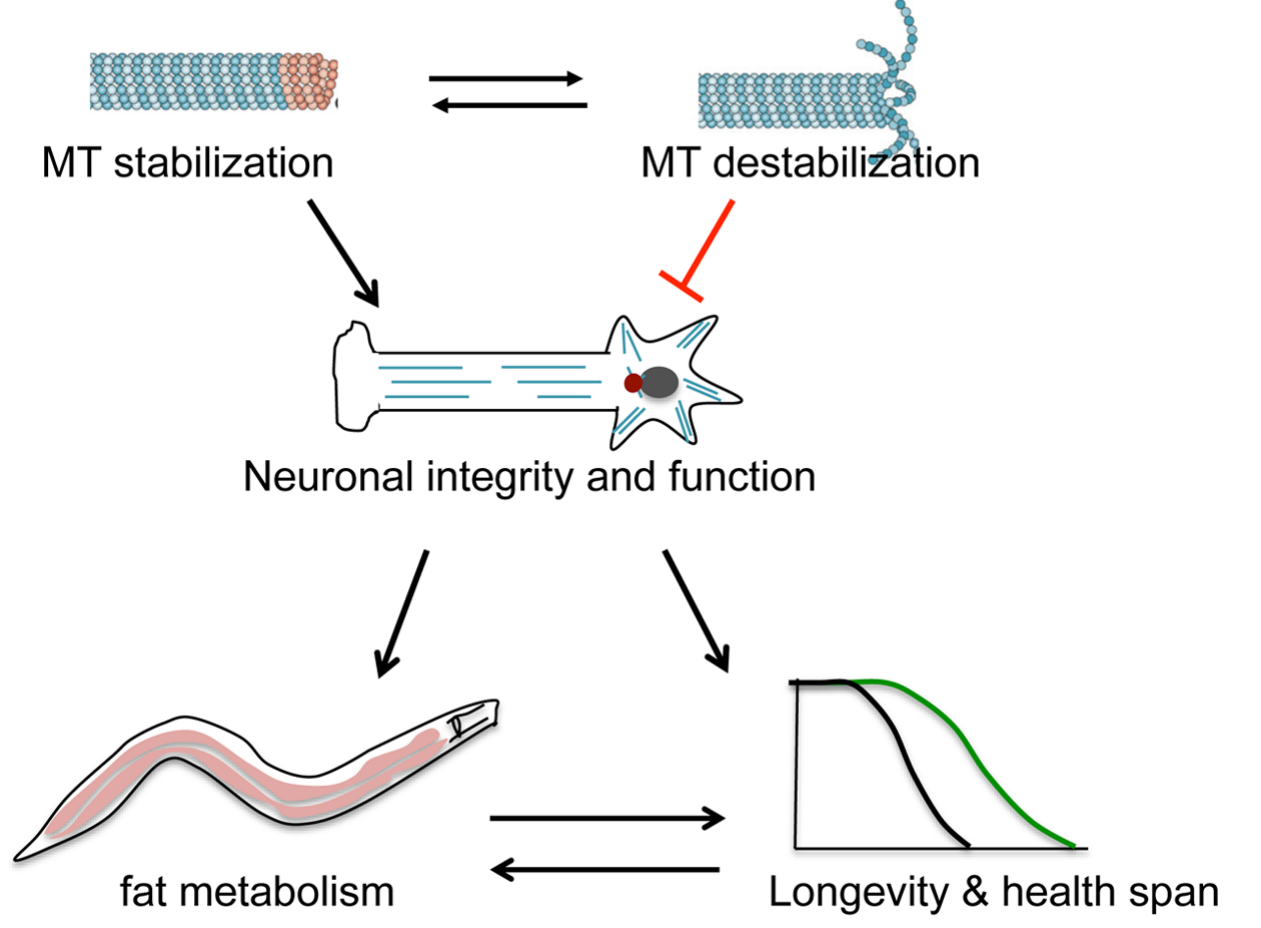
**Working model.** Neuronal microtubule (MT) status can affect neuronal integrity and function to impact organismal aging.

Loss-of-function mutants for *ptrn-1* and *hdac-6* were also studied [6]. PTRN-1 promotes MT stabilization by protecting MT minus ends, and HDAC-6 destabilizes MTs through tubulin de-acetylation. The lifespan changes seen with *ptrn-1* and *hdac-6* mutants were similar to the lifespan changes seen with *ptl-1* and efa-6 mutants respectively, providing more support that a preference for microtubule stabilization over destabilization within neurons promotes increased mean lifespan. Further, DAF-16/FOXO, the downstream transcription factor in the insulin/insulin-like growth factor signaling (IIS) pathway, is necessary for the increased mean lifespan seen in both *efa-6* and *hdac-6* mutants. To understand how the MT regulators contribute to animal lifespan, we examined the transcription profiles in the mutants with RNA-seq. Differentially expressed genes in MT regulator mutants were genes for lipid metabolism and lipid transport respectively. Consistent with the gene expression changes, altered fat storage was observed in the microtubule regulator mutants, suggesting that these changes may also contribute to the changes in lifespan. Taken together, these observations lend support to the concept that MT status impact neuronal function, which can influence lipid metabolism in other tissues and organismal longevity (Figure 1).

However, future studies are needed for questions remained. Changes in lipid metabolism in MT mutants might contribute to the lifespan phenotype, but could also be a correlation or consequence of altered longevity in MT regulator mutants, as many pathways that regulate longevity affect lipid biology. It is still unclear whether a particular subset of neurons may play a larger role in the observed effects than other neurons. There is the potential that differentially regulated microtubule dynamics within different subsets of neurons result in different effects on mean lifespan and that the net effect when microtubule stabilization is favored in all neurons results in an increase in mean lifespan. Further research should examine age-associated changes in microtubule regulator activity in different subsets of neurons to determine if certain neuronal subsets are more important in affecting lifespan and whether there are differential effects on lifespan depending on affected neuronal type. Because the effects of the microtubule regulation on lifespan are dependent on DAF-16, another course of future study would be to determine the precise mechanism(s) by which DAF-16 influences these microtubule regulators during normal aging as well as alterations that may occur in models of disease states in which altered microtubule dynamics are known to play a role.
